# Tegaserod for Irritable Bowel Syndrome With Constipation in Women Younger Than 65 Years Without Cardiovascular Disease: Pooled Analyses of 4 Controlled Trials

**DOI:** 10.14309/ajg.0000000000001313

**Published:** 2021-05-25

**Authors:** Eric D. Shah, Brian E. Lacy, William D. Chey, Lin Chang, Darren M. Brenner

**Affiliations:** 1 Section of Gastroenterology and Hepatology, Dartmouth-Hitchcock Medical Center, Lebanon, New Hampshire, USA;; 2 Division of Gastroenterology & Hepatology, Mayo Clinic, Jacksonville, Jacksonville, Florida, USA;; 3 University of Michigan Health System, Ann Arbor, Michigan, USA;; 4 David Geffen School of Medicine at UCLA, Los Angeles, California, USA;; 5 Division of Gastroenterology and Hepatology Northwestern University—Feinberg School of Medicine, Chicago, Illinois, USA.

## Abstract

**INTRODUCTION:**

Tegaserod was the first US Food and Drug Administration–approved drug for irritable bowel syndrome with constipation (IBS-C) in women and was recently reapproved for use. Recognizing that clinical trials were performed almost 20 years ago, we performed an integrated analysis on patient-reported outcomes relevant to current practice including previously unpublished data.

**METHODS:**

Data from 4 12-week, randomized, placebo-controlled trials evaluating tegaserod 6 mg b.i.d. in patients with IBS-C were pooled. We analyzed 2 groups: all women (overall population) and women younger than 65 years without a history of cardiovascular ischemic events (indicated population). The primary end point was subjective global assessment of IBS-C symptom relief. Responders rated themselves as “considerably” or “completely” relieved ≥50% of the time or at least “somewhat relieved” 100% of the time over the last 4 weeks.

**RESULTS:**

The overall and indicated populations included 2,939 (tegaserod [n = 1,478]; placebo [n = 1,461]) and 2,752 (tegaserod [n = 1,386]; placebo [n = 1,366]) participants, respectively. The pooled odds ratios (95% confidence interval) for clinical response during the last 4 weeks in the overall and indicated populations with tegaserod were 1.37 (1.18, 1.59; *P* < 0.001) and 1.38 (1.18, 1.61; *P* < 0.001). In the overall and indicated populations, clinical response rates for tegaserod during the last 4 weeks were 43.3% and 44.1% versus 35.9% and 36.5% with placebo (*P* < 0.001). Adverse events were similar between groups. No significant cardiovascular events related to tegaserod were observed in patients with ≤1 cardiac risk factor.

**DISCUSSION:**

Tegaserod 6 mg b.i.d. reduced IBS-C symptoms in overall and US Food and Drug Administration–indicated populations (women aged <65 years with no history of cardiovascular ischemic events).

## Graphical abstract

**Figure:**
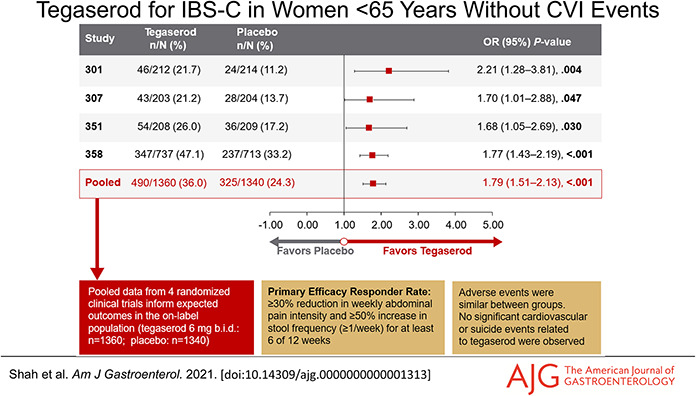

## INTRODUCTION

Irritable bowel syndrome with constipation (IBS-C) is a disorder of gut–brain interaction (previously called a functional gastrointestinal disorder) that affects 30% of the approximately 5%–9% of adults in the United States (US) diagnosed with IBS (1,2). IBS-C is defined by the Rome IV criteria as recurrent abdominal pain associated with defecation and/or a change in stool frequency or form (1). Symptoms of abdominal bloating and distention occur in most patients with IBS, although they are not required to make a diagnosis of IBS (1,3,4). IBS is associated with substantial medical costs, impaired functioning, and reduced quality of life (QOL) (5–9). In a US population–based survey of 1,667 individuals meeting symptom criteria for IBS-C, the most common symptoms reported by patients with IBS-C were abdominal pain (83%), bloating (78%), and straining (75%) (9). The most bothersome symptoms of IBS-C identified in a separate representative US population–based survey of more than 10,000 individuals accounting for variations in age, sex, and ethnicity were constipation, straining during bowel movements, gas pain, and abdominal discomfort (7).

There is no validated treatment algorithm for IBS-C. Treatments vary widely based on predominant symptom(s), cost, previous therapies used, risk/benefit analysis, and mechanisms of action. Common empirical treatments include dietary and behavioral changes, fiber products, prebiotic and probiotic supplements, osmotic agents, antispasmodics, and neuromodulators (4,10,11). However, the efficacy of most of these treatments has not been assessed in high-quality randomized controlled trials (RCT) in patients with IBS-C. Secretagogues such as linaclotide (Linzess), plecanatide (Trulance), tenapanor (Ibsrela), and lubiprostone (Amitiza) are US Food and Drug Administration (FDA) approved and commonly used.

Serotonin (5-HT), including the 5-HT_4_ receptor subtype, has been shown to be important in the pathophysiology of IBS-C because of its role in both gastrointestinal smooth muscle relaxation and contraction and visceral perception (12). It has been suggested that IBS-C may be related to reduced cellular release of 5-HT in the gastrointestinal tract. Tegaserod (Zelnorm; Alfasigma, USA, Inc, Covington, LA) is a 5-HT_4_ receptor agonist originally approved by the US FDA in 2002 for the short-term treatment of women with IBS-C (13,14). Initial concerns over possible cardiovascular ischemic (CVI) adverse events (AEs) in patients who did not fall within current regulatory guidelines led to the voluntary withdrawal of tegaserod in 2007 (15,16). Tegaserod was reintroduced in 2019 and is approved at a dose of 6 mg twice daily (b.i.d.) for treating IBS-C in women younger than 65 years without a history of CVI events (angina, myocardial infarction, transient ischemic attack, or stroke) (14). Tegaserod is the only 5-HT_4_ agonist indicated in the United States for treating IBS-C that specifically targets 5-HT receptors. Activation of 5-HT_4_ receptors located presynaptically on cholinergic neurons in the gastrointestinal tract stimulates acetylcholine release, leading to improvements in both gastrointestinal motility and visceral sensation and accelerating gastrointestinal transit (17–20). Individual studies conducted with tegaserod in different patient populations and at different doses have reported significant improvements in key symptoms of IBS-C with a good tolerability profile (21–23). This analysis is the first to summarize the efficacy and safety data on tegaserod in the US FDA-approved treatment population (women younger than 65 years with no history of CVI events) relevant to contemporary clinical practice in managing one of the most common referrals to gastroenterologists. Analyses were conducted using data aggregated from 4 RCTs (including previously unpublished data) that evaluated the efficacy and safety of tegaserod 6 mg b.i.d. in the treatment of IBS-C in all women and in patients that reflect the currently indicated population.

## METHODS

### Study design

Data from 4 separate 12-week RCTs (studies 301, 307, 351, and 358) that enrolled men and women with a diagnosis of IBS-C who had IBS symptoms for ≥3 months were pooled, including unpublished data from study 351 (21,22,24). In all 4 studies, patients underwent a 4-week treatment-free observational baseline period before being randomly assigned to 12 weeks of treatment with tegaserod or placebo, both administered twice daily. For one trial (study 358), the 12-week treatment period was followed by a 4-week withdrawal period during which patients received no study medication (22). Additional details of the study designs are described in Supplemental Table 1 (see Supplementary Digital Content 1, http://links.lww.com/AJG/C32). For all outcomes, we report pooled analyses of data for all women receiving tegaserod 6 mg b.i.d. or placebo. Unfortunately, pooled analyses of these outcomes are not available for the total study population that included men in the original clinical trials, recognizing that tegaserod is not approved for use in men. In the individual studies, the proportions of male participants ranged from 0% (study 358) to 17% (study 301). Consistent with the current US FDA labeling, our efficacy and safety analyses focused on data from women younger than 65 years with no history of CVI events who received tegaserod 6 mg b.i.d. or placebo, referred to henceforth as the indicated population.

Secondary efficacy end points were analyzed in a slightly different population that included women with no history of CVI events without any restrictions on age receiving tegaserod 6 mg b.i.d. or placebo (heretofore referred to as women with low cardiovascular [CV] risk). For these secondary efficacy end point analyses, 99.8% of patients were younger than 65 years; the remaining 0.2% of patients could not be excluded based on the available data. In addition to excluding women with histories of CVI events, our analyses on secondary efficacy end points also excluded those with the following CV risk factors: active smoking, history of hypertension, hyperlipidemia, diabetes mellitus, and obesity (body mass index >30 kg/m^2^).

### Assessments

Subjective global assessment (SGA) of relief is a single measure encompassing abdominal pain/discomfort, altered bowel function, and overall well-being. This measure, which has been validated in populations with IBS, was considered the standard assessment of symptoms for IBS trials in the past (21,25). Patients completed the SGA of relief weekly rating their overall well-being and symptoms of abdominal discomfort/pain and altered bowel habits during the last week as completely relieved, considerably relieved, somewhat relieved, unchanged, or worse in relation to the way they usually felt before entering the study.

Daily assessments of abdominal pain and discomfort over the last 24 hours were assessed with the SGA of abdominal pain and discomfort, a self-administered visual analog scale (100 mm in length), with severity descriptors of absent, very mild, mild, moderate, severe, and very severe. Patients also recorded the following information on a daily basis: number of bowel movements; stool consistency (1, watery; 2, loose; 3, somewhat loose; 4, neither loose nor hard; 5, somewhat hard; 6, hard; and 7, very hard); and severity of abdominal pain and discomfort and severity of bloating (for both: 0, none; 1, very mild; 2, mild; 3, moderate; 4, severe; and 5, very severe).

For all studies, the safety assessments included AE monitoring, physical examinations, vital signs, pregnancy screening, standard laboratory evaluations, and electrocardiograms.

### End points and statistical analyses

Based on guidance by the US FDA when tegaserod was originally approved, primary efficacy end points for all included studies were an SGA of relief of IBS-C symptoms, where responders were defined as rating themselves “considerably” or “completely” relieved ≥50% of the time or at least “somewhat relieved” 100% of the time (50%/100% SGA) over the first month and the last 4 weeks of the 12-week studies (26). Under more recent review by the US FDA leading to approval based on the original clinical trial data, patients were categorized as responders using a composite end point of a ≥30% reduction in weekly abdominal pain intensity and a ≥50% increase in stool frequency (≥1/wk) for at least 6 of the 12 weeks of the study (coprimary end point) (27). Secondary efficacy end points included the proportions of patients experiencing complete, considerable, or somewhat relief of their IBS symptoms and the proportion of patients with abdominal pain/discomfort, bloating, and bowel movement treatment responses (≥2-point improvement from baseline on a 7-point Likert scale) at 12 weeks. As a supportive analysis, components of the responder rate for SGA of relief at the end point (patients completely relieved or considerably relieved ≥50% at the end point and patients at least somewhat relieved 100% of the time at the end point) from individual studies (301, 307, 351, and 358) were summarized for the total patient population.

Efficacy end points were analyzed in the intent-to-treat (ITT) population, which included all patients randomized to study treatment. Efficacy outcomes are reported as odds ratios (OR) with 95% confidence intervals (CI). Differences in treatment response rates were calculated using Cochran-Mantel-Haenszel asymptotic estimates, with *P* values based on a Cochran-Mantel-Haenszel χ^2^ test controlled by pooled investigator site. Missing data were excluded from the analyses.

Safety end points were analyzed using the safety population, which consisted of all randomized patients who received ≥1 dose of study medication and had ≥1 safety assessment. Treatment-emergent AEs (TEAEs), severe TEAEs, TEAEs leading to discontinuation, and serious TEAEs were summarized using descriptive statistics. As an additional supportive analysis, cases with confirmed adjudicated cardiovascular events and major adverse cardiac events (MACEs) identified during adjudication of 29 placebo-controlled studies in any indication with duration ≥4 weeks were reported.

## RESULTS

### Patients

**Figure 1.**
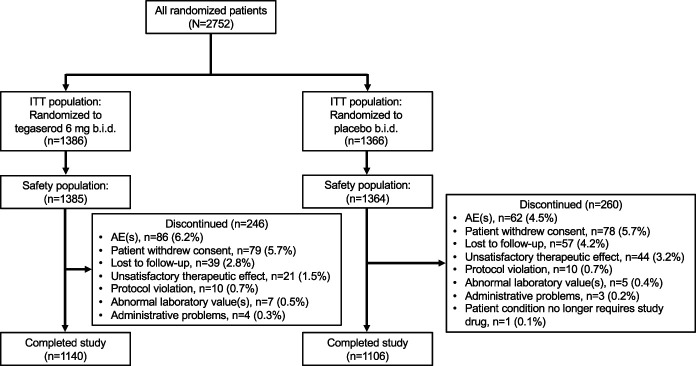
Patient disposition (indicated population). The indicated population was defined as women younger than 65 years with no history of CVI events who received tegaserod 6 mg b.i.d. or placebo. AE, adverse event; b.i.d., twice daily; CVI, cardiovascular ischemic; ITT, intent-to-treat.

Among all women, the ITT population for the primary and secondary efficacy analyses included 2,939 patients randomized to tegaserod (n = 1,478 [study 301, n = 244; study 307, n = 233; study 351, n = 234; and study 358, n = 767]) or placebo (n = 1,461 [study 301, n = 240; study 307, n = 234; study 351, n = 235; study 358, n = 752]) (see Supplemental Figure 1, Supplementary Digital Content 1, http://links.lww.com/AJG/C28). The ITT population for the primary efficacy analysis in the indicated population included 2,752 patients randomized to tegaserod (n = 1,386 [study 301, n = 219; study 307, n = 209; study 351, n = 220; study 358, n = 738]) or placebo (n = 1,366 [study 301, n = 217; study 307, n = 212; study 351, n = 218; study 358, n = 719]) (Figure 1). The ITT population for secondary efficacy analyses conducted in women with low CV risk included 1,122 patients in the tegaserod group and 1,079 in the placebo group. Among all women, the safety population included 1,477 patients randomized to tegaserod and 1,459 randomized to placebo. The safety analyses that were conducted in the indicated population included 1,385 patients randomized to tegaserod and 1,364 randomized to placebo. Two additional patients discontinued treatment in study 301, but data could not be obtained to ascertain the reason for study withdrawal. Patient disposition among all women is described in Supplemental Figure 1 (see Supplementary Digital Content 1, http://links.lww.com/AJG/C28). For the indicated population, the number (%) of patients who discontinued the study early was 246 (17.7%) in the tegaserod group and 260 (19.0%) in the placebo group (Figure 1). Overall, 1,140 (82.3%) patients receiving tegaserod and 1,106 (81.0%) receiving placebo completed the trials. Baseline demographics and duration of symptoms were similar between treatment groups (Table 1).

**Table 1.**
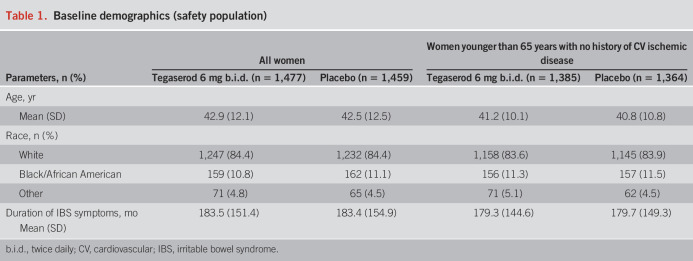
Baseline demographics (safety population)

### Efficacy analyses

#### Primary efficacy end points.

**Figure 2.**
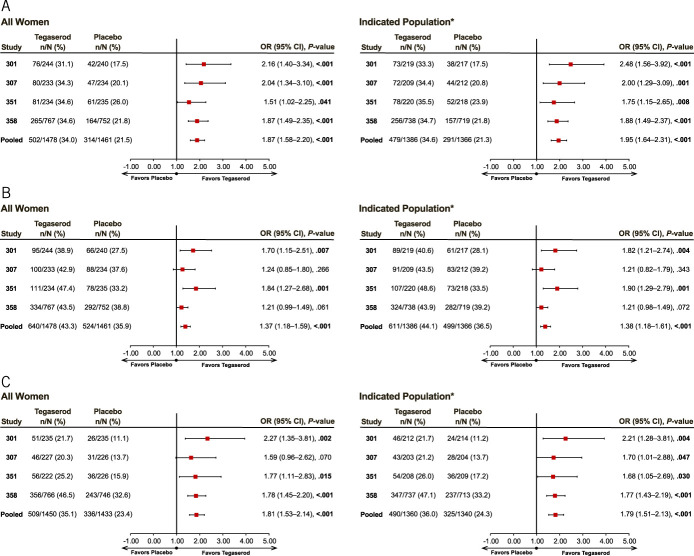
(**a**) 50%/100% SGA responders, month 1. 50%/100% SGA responders were defined as subjects rating themselves considerably or completely relieved ≥50% of the time or at least somewhat relieved 100% of the time. *The indicated population was defined as women younger than 65 years with no history of CVI events who received tegaserod 6 mg b.i.d. or placebo. (**b**) 50%/100% SGA responders, last 4 visits. 50%/100% SGA responders were defined as subjects rating themselves considerably or completely relieved ≥50% of the time or at least somewhat relieved 100% of the time. *The indicated population was defined as women younger than 65 years with no history of CVI events who received tegaserod 6 mg b.i.d. or placebo. (**c**) Abdominal pain/stool frequency responder, 12 weeks. Response defined as ≥30% reduction in weekly abdominal pain intensity and ≥50% increase in stool frequency (≥1/wk) for at least 6 of 12 weeks. *The indicated population was defined as women younger than 65 years with no history of CVI events who received tegaserod 6 mg b.i.d. or placebo. b.i.d., twice daily; CI, confidence interval; CVI, cardiovascular ischemic; OR, odds ratio; SGA, subjective global assessment.

The primary efficacy analyses included data from all women and from women younger than 65 years without a history of CVI events (indicated population). Among all women, the pooled OR (95% CI) for achieving 50%/100% SGA response was 1.87 (1.58, 2.20; *P* < 0.001; number needed to treat (NNT) [95% CI], 9 [7, 11]) at 1 month (Figure 2A) and 1.37 (1.18, 1.59; *P* < 0.001; NNT [95% CI], 14 [10, 26]) during the last 4 weeks (Figure 2B). Corresponding values in the indicated population were 1.95 (1.64, 2.31; *P* < 0.001; NNT [95% CI], 8 [7, 11]) at 1 month (Figure 2A) and 1.38 (1.18, 1.61; *P* < 0.001; NNT [95% CI], 14 [9, 25]) during the last 4 weeks (Figure 2B). The 50%/100% SGA response rates during the last 4 weeks among all women were 43.3% with tegaserod 6 mg b.i.d. compared with 35.9% for placebo (difference [95% CI], 7.4% [3.9%, 11.0%; *P* < 0.001]) and in the indicated population were 44.1% with tegaserod 6 mg b.i.d. compared with 36.5% for placebo (difference [95% CI], 7.7% [4.0%, 11.3%; *P* < 0.001]). Significant benefits were also observed regarding the composite abdominal pain and stool frequency response using tegaserod compared with placebo in all women (pooled OR [95% CI], 1.81 [1.53, 2.14]; *P* < 0.001; NNT [95% CI], 9 [7, 13]) and in the indicated population (pooled OR [95% CI], 1.79 [1.51, 2.13]; *P* < 0.001; NNT [95% CI], 9 [7, 13]) (Figure 2C). The US FDA composite end point of a ≥30% reduction in weekly abdominal pain intensity and a ≥50% increase in stool frequency (≥1/wk) for at least 6 of the 12 weeks of treatment was achieved by 35.1% and 23.4% of all women receiving tegaserod and placebo, respectively (difference [95% CI], 11.4% [8.2%, 14.7%]; *P* < 0.001). Corresponding values in the indicated population were 36.0% of tegaserod-treated patients versus 24.3% of those receiving placebo (difference [95% CI], 11.5% [8.1%, 14.8%]; *P* < 0.001).

#### Secondary efficacy end points.

**Figure 3.**
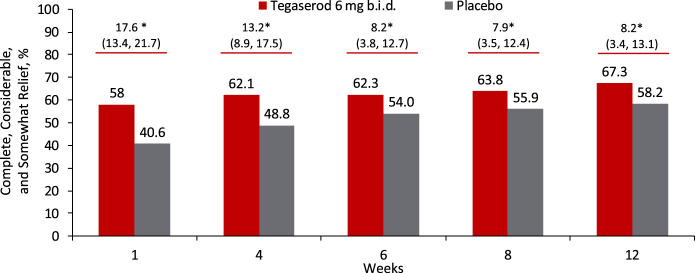
Complete, considerable, and somewhat relief of IBS symptoms at each week (population with low CV risk). *P < 0.001. The population with low CV risk was defined as women with no history of CVI events without any restrictions on age receiving tegaserod 6 mg b.i.d. or placebo. b.i.d., twice daily; CV, cardiovascular; CVI, cardiovascular ischemic; IBS, irritable bowel syndrome.

**Figure 4.**
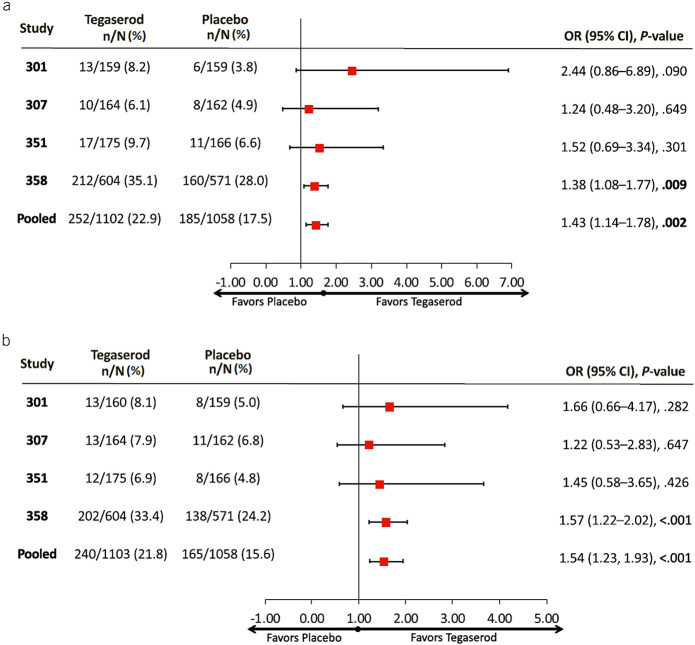
(**a**) Abdominal pain responder, 12 weeks (population with low CV risk). Abdominal pain response defined as a ≥2-point improvement on a 7-point Likert scale. The population with low CV risk was defined as women with no history of CVI events without any restrictions on age receiving tegaserod 6 mg b.i.d. or placebo. (**b**) Bloating responder, 12 weeks (population with low CV risk). Bloating response defined as a ≥2-point improvement on a 7-point Likert scale. The population with low CV risk was defined as women with no history of CVI events without any restrictions on age receiving tegaserod 6 mg b.i.d. or placebo. b.i.d., twice daily; CI, confidence interval; CVI, cardiovascular ischemic; OR, odds ratio.

**Figure 5.**
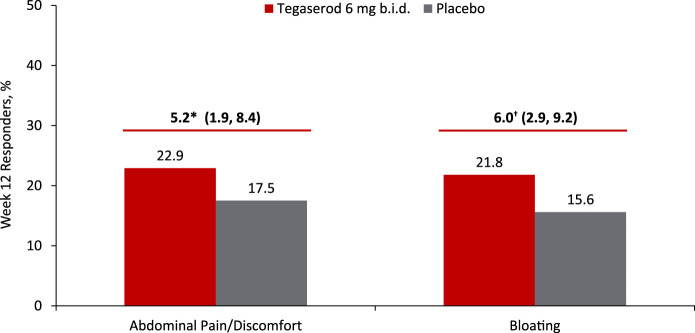
Abdominal pain/discomfort and bloating responders, 12 weeks (population with low CV risk). *P < 0.01; †*P* < 0.001. Response was defined as a ≥2-point improvement on a 7-point Likert scale. The population with low CV risk was defined as women with no history of CVI events without any restrictions on age receiving tegaserod 6 mg b.i.d. or placebo. b.i.d., twice daily; CVI, cardiovascular ischemic.

Secondary efficacy end points were assessed in all women receiving tegaserod 6 mg b.i.d. or placebo. Based on available data, these analyses were additionally performed in the population of low CV risk women without any restrictions on age, recognizing that 99.8% of patients in this analysis were younger than 65 years. Among all women, a significantly higher proportion of patients experienced complete, considerable, or somewhat relief of their IBS symptoms in the tegaserod group versus the placebo group at each week, from week 1 through week 12 (all *P* ≤ 0.002; see Supplemental Figure 2, Supplementary Digital Content 2, http://links.lww.com/AJG/C29). As early as week 1, a significantly higher proportion of low CV risk women experienced complete, considerable, or somewhat relief of their IBS symptoms in the tegaserod group compared with the placebo group (*P* < 0.001 for all comparisons; Figure 3), and this was maintained through week 12. The percentage of low CV risk women who experienced complete, considerable, or somewhat relief of their IBS symptoms at week 12 was 67.3% in the tegaserod group and 58.2% in the placebo group (difference [95% CI]: 8.2% [3.4, 13.1]; OR [95% CI], 1.43 [1.16, 1.76]; *P* < 0.001; Figure 3). A significantly higher proportion of patients also met response criteria at week 12 for the secondary end points of abdominal pain/discomfort (all women: tegaserod, 22.4%; placebo, 17.6%; difference [95% CI], 4.2% [1.9, 7.5]; OR [95% CI], 1.38 [1.14, 1.67]; *P* < 0.001; NNT [95% CI], 22 [14, 53]; see Supplemental Figure 3A, Supplementary Digital Content 3, http://links.lww.com/AJG/C30; women with low CV risk: tegaserod, 22.9%; placebo, 17.5%; difference [95% CI], 5.2% [1.9, 8.4]; *P* = 0.002; OR [95% CI], 1.43 [1.14, 1.78]; *P* = 0.002; NNT [95% CI], 20 [12, 52]; Figure 4a and Figure 5), bloating (all women: tegaserod, 20.8%; placebo, 16.1%; difference [95% CI], 4.6% [1.8, 7.3]; OR [95% CI], 1.39 [1.14, 1.69]; *P* = 0.001; NNT [95% CI], 22 [14, 55]; see Supplemental Figure 3B, Supplementary Digital Content 4, http://links.lww.com/AJG/C31; women with low CV risk: tegaserod, 21.8%; placebo, 15.6%; difference [95% CI], 6.0% [2.9, 9.2]; OR [95% CI], 1.54 [1.23, 1.93]; *P* < 0.001; NNT [95% CI], 17 [11, 35]; Figure 4b and Figure 5), and bowel movements (all women: tegaserod, 49.4%; placebo, 31.9%; difference [95% CI], 17.3% [13.8, 20.8]; OR [95% CI], 2.09 [1.80, 2.44]; *P* < 0.001; NNT [95% CI], 6 [5, 8]; women with low CV risk: tegaserod, 49.7%; placebo, 33.1%; difference [95% CI], 16.2% [12.1, 20.3]; OR [95% CI], 1.99 [1.67, 2.38]; *P* < 0.001; NNT [95% CI], 7 [5, 9]).

#### Supportive efficacy end points.

With the exception of the criterion of at least somewhat relieved 100% of the time at end point in studies 301 and 351, differences between tegaserod 6 mg b.i.d. and placebo were either unavailable or not statistically significant (see Supplemental Table 2, Supplementary Digital Content 5, http://links.lww.com/AJG/C32).

### Safety analyses

Safety analyses were performed in all women and the indicated population. At least 1 TEAE was reported by 65.9% of patients in the tegaserod group and 64.0% in the placebo group among all women and by 65.8% of patients in the tegaserod group and 63.3% in the placebo group in the indicated population. Among all women, the most frequent TEAEs were headache (tegaserod, 13.7%; placebo, 12.2%), abdominal pain (tegaserod, 12.5%; placebo, 11.5%), diarrhea (tegaserod, 8.7%; placebo, 4.0%), and nausea (tegaserod, 8.0%; placebo, 6.8%) (Table 2). Similarly, in the indicated population, headache was the most frequent TEAE in both treatment groups (tegaserod, 14.2%; placebo, 12.1%), followed by gastrointestinal TEAEs such as abdominal pain (tegaserod, 12.3%; placebo, 10.9%), diarrhea (tegaserod, 8.6%; placebo, 3.9%), and nausea (tegaserod, 8.0%; placebo, 6.8%) (Table 2). The incidence of severe TEAEs was similar in the tegaserod and placebo groups (all women, 17.5% vs 16.2%; indicated population, 17.5% vs 15.8%). The most common class of severe TEAEs was gastrointestinal disorders, particularly abdominal pain (all women: tegaserod, 5.1%; placebo, 4.2%; indicated population: tegaserod, 5.1%; placebo, 4.0%) and diarrhea (all women: tegaserod, 2.4%; placebo, 0.9%; indicated population: tegaserod, 2.4%; placebo, 0.8%).

**Table 2.**
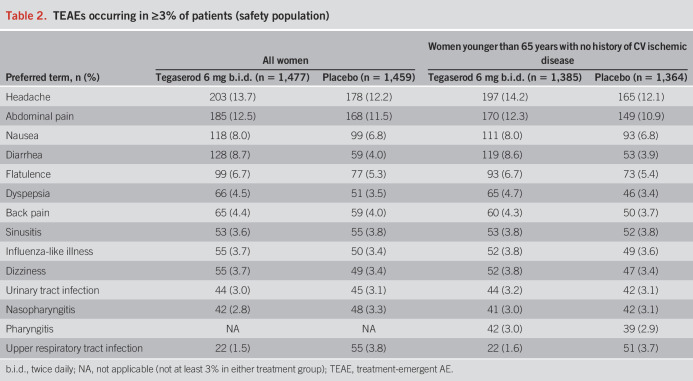
TEAEs occurring in ≥3% of patients (safety population)

For all women, the percentage who experienced a TEAE leading to discontinuation was 6.6% in the tegaserod group and 5.3% in the placebo group. Among patients in the indicated population receiving tegaserod, 6.2% experienced a TEAE leading to discontinuation; this rate was slightly higher than among those receiving placebo (4.8%). Gastrointestinal symptoms, including abdominal pain (all women: tegaserod, 1.5% placebo, 1.2%; indicated population: tegaserod, 1.4%; placebo, 1.0%) and diarrhea (all women: tegaserod, 1.4% placebo, 0.2%; indicated population: tegaserod, 1.4%; placebo, 0.1%), were the most common class of TEAEs leading to discontinuation.

Among all women, 1.4% of tegaserod-treated patients and 0.9% of those receiving placebo experienced at least 1 serious TEAE. In the indicated population, serious TEAEs were experienced by 19 patients (1.4%) in the tegaserod group and 12 patients (0.9%) in the placebo group. Only 1 patient (0.1%) who received tegaserod experienced a serious CV TEAE (coronary artery disease, occurring on day 12 of treatment). This patient, a woman included in the indicated population, was found to have severe coronary artery disease in the left main, circumflex, and right coronary arteries. As a result, the patient underwent bypass surgery on day 17 after which a final electrocardiogram found no clinically significant abnormalities. The investigator did not consider this event as related to study medication. Among all women, 1 patient (0.1%), who was also included in the indicated population, had a suicide attempt while receiving placebo, while no patients had a suicide attempt while receiving tegaserod.

Supplemental Table 3 (see Supplementary Digital Content 5, http://links.lww.com/AJG/C32) summarizes cases with confirmed adjudicated cardiovascular events and MACE identified during adjudication of 29 placebo-controlled tegaserod studies in any indication with durations of ≥4 weeks. In the first adjudication, the number and percentage of tegaserod-treated patients with MACE was low among all patients (n = 7, 0.06%), even lower among women (n = 5, 0.05%), and zero in the indicated population. The same pattern was generally observed for the second adjudication, although the percentage of tegaserod-treated patients with MACE was 0.03% for all patients and for women only.

## DISCUSSION

In these pooled analyses, tegaserod effectively reduced a range of symptoms related to IBS-C among all women and in women younger than 65 years without a history of CVI events (indicated population). Improvements were observed in overall SGA response and in individual symptoms of abdominal pain/discomfort, bloating, and stool frequency. More importantly, using the recent US FDA-suggested guidelines that define a treatment response, which were not available at the time of the initial studies, patients treated with tegaserod were more likely than those in the placebo group to experience a ≥30% reduction in weekly abdominal pain intensity and a ≥50% increase in stool frequency (≥1/wk) for ≥6 of the 12 weeks of study treatment. Effects were rapid, with significant relief of IBS symptoms as early as week 1 and significant benefit over placebo in odds of achieving 50%/100% SGA response by month 1. Benefits were sustained over 12 weeks of treatment. In addition, tegaserod improved other important symptoms of IBS-C, including abdominal pain/discomfort, bowel movements, and bloating in all women with IBS-C and in those women who had no history of CVI events or CV risk factors and were primarily (99.8%) younger than 65 years.

A network meta-analysis of data from the 12-week trials in patients with IBS-C compared tegaserod 6 mg b.i.d. (based on studies 301, 351, and 358) with other approved pharmacotherapies for IBS-C, including linaclotide 290 μg q.d., lubiprostone 8 μg b.i.d., plecanatide 3 and 6 mg b.i.d., and tenapanor 50 mg b.i.d (28). The relative risk of failure (as opposed to the likelihood of achieving response, as calculated in this analyses) to achieve the US FDA-recommended efficacy end point of a ≥30% reduction in abdominal pain and an increase of ≥1 complete spontaneous bowel movement/week from baseline for ≥50% of the weeks of study treatment was calculated as 0.85 (95% CI, 0.80, 0.91) for tegaserod, thus demonstrating a significant benefit in abdominal pain and stool frequency response, as was observed in this analysis. Tegaserod trials differed from the more recent US FDA guidance in that the original IBS-C trials combined pain intensity and discomfort into a single end point and did not define a spontaneous bowel movement regarding complete evacuation (15). Linaclotide was ranked as the most effective; however, indirect comparisons revealed no significant differences between medications. By contrast, a separate meta-analysis of the same clinical trials found no difference in the ORs for efficacy end points between the US FDA–approved secretagogues (29). The true differences among IBS-C treatments remain unknown because of the lack of head-to-head comparisons in this patient population. Ultimately, the recently published American College of Gastroenterology guidelines provide recommendations on the use of the US FDA–approved medications for treating IBS, including tegaserod (30).

Safety and tolerability analyses showed a low incidence of TEAEs among all women and in women with IBS-C younger than 65 years with no history of CVI events who received tegaserod. In both patient populations, headaches and gastrointestinal symptoms were the most common TEAEs and did not differ substantially in frequency between patients who received tegaserod or placebo. The rate of treatment discontinuations related to diarrhea was very low (all women: 1.4% with tegaserod and 0.2% with placebo; indicated population: 1.4% with tegaserod and 0.1% with placebo). By contrast, for the guanylate cyclase C agonist linaclotide, diarrhea was the most frequent TEAE, occurring in up to 20% of patients receiving linaclotide and 3% receiving placebo, and was the most common TEAE leading to discontinuation (5% vs 1%) (31). In 2 clinical trials, during double-blind treatment periods of 12 and 26 weeks, respectively, the locally acting sodium/hydrogen exchanger 3-inhibitor tenapanor was associated with a 15%–16% prevalence of diarrhea (vs 2%–4% in placebo groups) and a 6.5% discontinuation rate due to diarrhea (vs 0.7% in patients receiving placebo) (32). Notably, the above-described network meta-analysis found that diarrhea was less likely with tegaserod than with either linaclotide or tenapanor (28). Based on product labeling, the prevalence of treatment discontinuation due to diarrhea in patients with IBS-C receiving the guanylate cyclase C agonist plecanatide (1.2%) (33) or the chloride channel activator lubiprostone (<1%) (34) was comparable with that of tegaserod.

Despite initial concerns over possible CVI AEs arising from clinical trials that led to voluntary withdrawal of tegaserod from the market in 2007 (15,16), subsequent matched case–control (35) and matched cohort studies (36) found no association between tegaserod and atherosclerotic CVD-related AEs or CVI AEs, respectively. Furthermore, several independent adjudications have reported that the risk of CV-related AEs is low among women younger than 65 years without a history of CVI events (angina, myocardial infarction, transient ischemic attack, or stroke) and no more than 1 CV risk factor (defined as active smoking, hypertension, hyperlipidemia, diabetes mellitus, age ≥55 years, obesity [body mass index >30 kg/m^2^]), recognizing that the indicated population for tegaserod actually comprises most of the patients with IBS in practice (37,38). These analyses are consistent with results of the supportive analysis presented in this publication (see Supplemental Table 3, Supplementary Digital Content 5, http://links.lww.com/AJG/C32), which identified no cases of MACE in the indicated population across 29 placebo-controlled studies and were used to support the reintroduction of tegaserod in 2019 for women younger than 65 years with no history of CVI events. In these analyses, only 1 patient receiving tegaserod (0.1%) experienced a serious CV TEAE (coronary artery disease) that was not considered related to study medication by the investigator. These data taken together demonstrate that tegaserod is safe in the indicated population, and a forthcoming publication will provide greater detail regarding the cardiovascular safety of tegaserod. However, it has been suggested that monitoring for signs of cardiovascular toxicity in clinical trials of tegaserod may not have been rigorous enough to detect subtle effects, leading to potential uncertainty about the risks associated with tegaserod (39). Nonetheless, the authors agreed that the absolute cardiac risk of tegaserod is likely low in the indicated population of patients who otherwise has a negligible risk.

Although the precise mechanism is unclear, the use of serotonin agonists and selective serotonin reuptake inhibitors has been associated with an increased risk of suicide (40,41). No patients in this analysis did a suicide attempt while receiving tegaserod, whereas 1 patient in the placebo group attempted suicide. In a review of all tegaserod randomized clinical trials, which included 10,951 patients treated with tegaserod and 6,236 treated with placebo, 8 (0.07%) patients receiving tegaserod and 1 (0.02%) receiving placebo experienced suicidal ideation/behavior (42). Two completed suicides were observed, both were patients with psychiatric histories and one was being treated with amitriptyline. The investigators did not consider either case to be related to treatment with tegaserod. The comprehensive safety analyses conducted with tegaserod concluded that there is low risk for suicidal behavior in patients treated with tegaserod (1.72 per 100,000 patient-years), recognizing that patients with chronic medical disorders such as IBS are more likely to experience psychological distress (15,43). Moreover, suicidal ideation/behavior in clinical trials was proportionately more frequent among patients receiving antidepressant medication (15). According to product labeling, patients taking tegaserod should be monitored for worsening of depression and emergence of suicidal thoughts and behaviors, especially during the first few months of treatment, and should be instructed to immediately discontinue tegaserod and contact their healthcare provider if they experience persistently worsening depression or emergent suicidal thoughts and behaviors (14).

Similar to all research studies, our analysis has potential limitations to consider. Because the studies included in the analyses were conducted approximately 20 years ago, the diagnostic criteria used have been eclipsed by more recent standards, and inclusion criteria differed from those required today; efficacy end points presented in this study approximate the current US FDA responder end points for IBS-C clinical trials and are the same as the end points used in evaluating the efficacy of tegaserod in its 2019 approval (26). Unfortunately, outcomes in men were not available to include in our analysis, recognizing that most patients enrolled in tegaserod trials were women and that tegaserod is not approved for use in men. Although the ORs for primary end points in the indicated population were both fairly high and statistically significant in favor of tegaserod, the net therapeutic gain in relation to placebo on both outcomes was relatively low (7.7% for 50%/100% SGA and 11.5% for abdominal pain and stool frequency), suggesting that a small subset of patients may have benefited most. Although attempts to identify the population most likely to benefit from tegaserod would be of clinical interest, such analyses are beyond the scope of this report. There were slight differences between our secondary efficacy analysis population (which excluded individuals with CV risk factors and included a small portion of patients aged 65 years or older [0.2% of the total secondary efficacy analysis population]) because of the availability of data. In addition, patients included in the analyses were predominantly (84%) White, limiting generalizability of findings to other race/ethnic groups. Despite these limitations, the strength of our analysis is the large overall population of patients and reporting of data consistent with the population described in the updated prescribing information for tegaserod. We also included data from a previously unpublished clinical trial (study 351).

Pooled analyses from 4 distinct prospective RCTs in patients with IBS-C demonstrated that tegaserod 6 mg b.i.d. effectively reduced overall and individual symptoms of IBS-C compared with placebo. Tegaserod significantly improved the core symptoms of IBS-C, including abdominal pain/discomfort, bloating, and bowel movement frequency in women younger than 65 years without a history of CVI events. Although headache was the most common TEAE, rates were similar between the tegaserod and placebo groups (12% vs 10%). Diarrhea led to drug discontinuation in a small percentage of patients. Given the potentially lower prevalence of treatment discontinuation because of diarrhea in patients with IBS-C treated with tegaserod relative to linaclotide and tenapanor, tegaserod may be considered an alternative for patients experiencing this AE with other treatments. Rates of serious AEs, including those related to CV risk factors or suicide events, were similarly low in the tegaserod and placebo groups. In conclusion, tegaserod 6 mg b.i.d. represents a safe and efficacious treatment option for IBS-C in women younger than 65 years with no history of CVI events.

## CONFLICTS OF INTEREST

**Guarantor of the article:** Eric D. Shah, MD, MBA.

**Specific author contributions:** Study design: All authors. Study investigator: N/A. Enrolled patients: N/A. Collection and assembly of data: N/A. Data analysis: All authors. Data interpretation: All authors. Manuscript preparation: All authors. Manuscript review and revisions: All authors. Final approval of manuscript: All authors.

**Financial support:** Dr. Shah's protected time is supported by the AGA Research Foundation's 2019 American Gastroenterological Association–Shire Research Scholar Award in Functional GI and Motility Disorders.

**Potential competing interests:** E.D.S.: reports travel conference registration reimbursement from Bausch Health outside the submitted work. B.E.L.: is a board member of the American College of Gastroenterology, the International Foundation of Functional GI Disorders, and the Rome Foundation. He reports personal fees from Alfasigma, outside the submitted work, and participates in scientific advisory boards for: Arena, Allakos, Salix/Bausch, Ironwood, Viver, and Urovant. W.D.C is a board member of: American College of Gastroenterology, GI Health Foundation, GI on Demand, International Foundation of Functional GI Disorders, and Rome Foundation; consultant for: Alfasigma, Allergan, Alnylam, Arena, Bayer, Biomerica, IM Health, Ironwood, QOL Medical, Orphomed, Phathom, Redhill, Ritter, Salix/Valeant, Takeda, Urovant, and Vibrant; and receives grant/research support from: Biomerica, Commonwealth Diagnostics International, QOL Medical, Salix, Urovant, and Vibrant. He holds stock or stock options in GI on Demand, Modify Health, and Ritter. L.C. is a board member of the American Gastroenterological Association and the Rome Foundation. She reports personal fees from Alfasigma, outside the submitted work, and participated in scientific advisory boards for: Arena, Ironwood, and Cosmo. She has served as a consultant for Allergan and Shire Takeda. She holds stock options for Modify Health. D.M.B. reports receiving consulting fees from Alfasigma. Dr. Brenner's research is also supported by an unrestricted gift from the Irene D. Pritzker Foundation.

**Study Highlights**

**WHAT IS KNOWN**

✓ Tegaserod is a 5-HT_4_ agonist and was the first US Food and Drug Administration–approved drug to treat irritable bowel syndrome with constipation (IBS-C) in women with an established clinical efficacy profile.
✓ Tegaserod was recently reapproved to treat IBS-C in women younger than 65 years without a history of cardiovascular ischemic events (angina, myocardial infarction, transient ischemic attack, or stroke).
✓ Recognizing that clinical trials were conducted almost 20 years ago, the efficacy and safety profile for the current indication was not known.

**WHAT IS NEW HERE**

✓ In the indicated population, tegaserod significantly increased the proportion of women with global IBS-C symptom relief compared with placebo.
✓ Tegaserod improved individual core symptoms of IBS-C, including bloating, abdominal pain/discomfort, and bowel movement frequency compared with placebo.
✓ Tegaserod 6 mg b.i.d. demonstrated an excellent safety profile in women younger than 65 years without a history of cardiovascular ischemic events.

## Supplementary Material

SUPPLEMENTARY MATERIAL
