# Supplementary material for: Tegaserod for Irritable Bowel Syndrome With Constipation in Women Younger Than 65 Years Without Cardiovascular Disease: Pooled Analyses of 4 Controlled Trials
Source: Am J Gastroenterol. 2021 May 25;116(8):1601–11. doi: 10.14309/ajg.0000000000001313 (PMC8315186; doi:10.14309/ajg.0000000000001313)

Supplemental Figure 1: Patient disposition (all women).

AE, adverse event; b.i.d., twice daily; ITT, intent-to-treat.

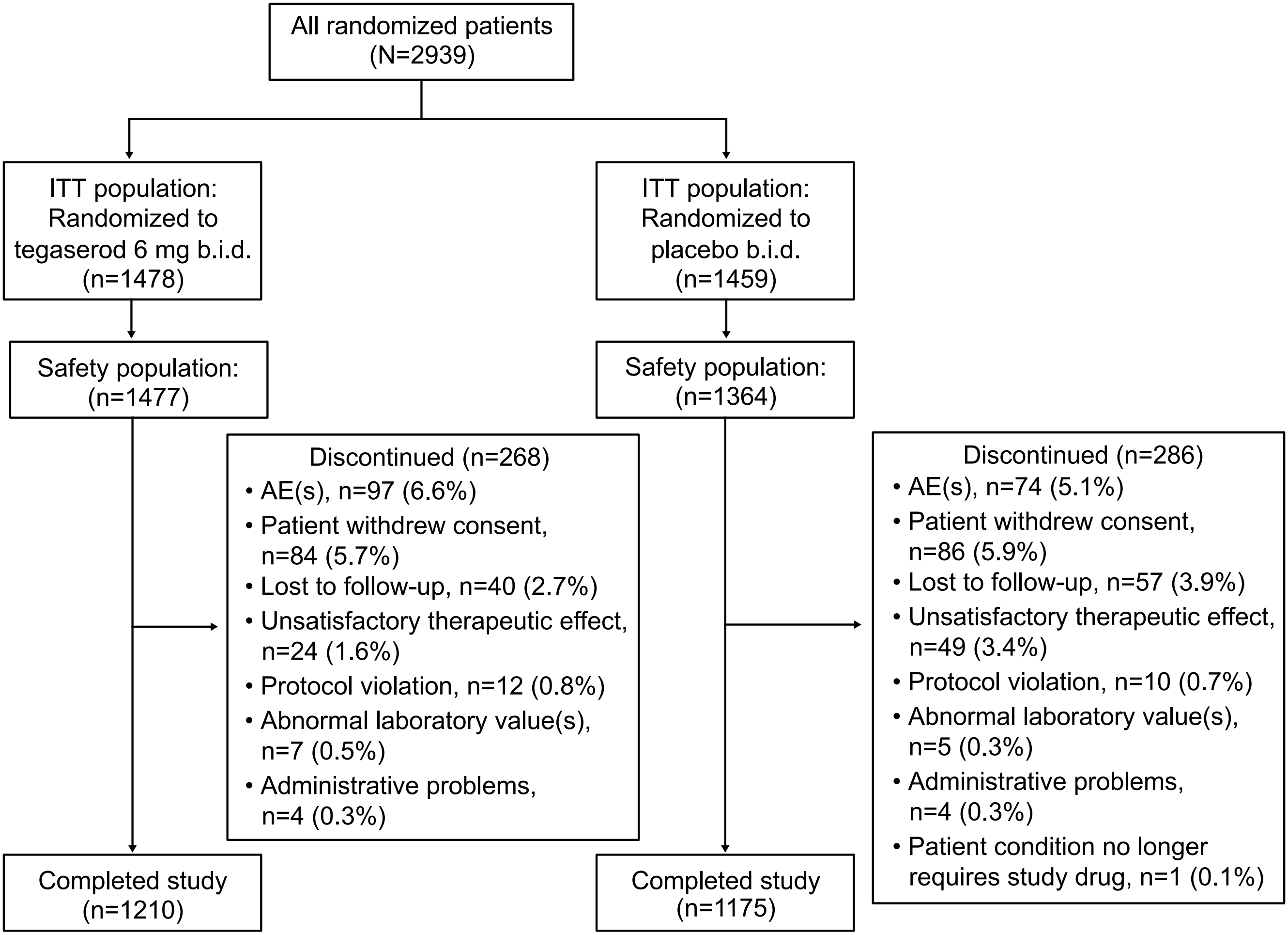

Supplement: SUPPLEMENTARY MATERIAL [file acg-116-1601-s001.pdf]
