# Supplementary material for: Tegaserod for Irritable Bowel Syndrome With Constipation in Women Younger Than 65 Years Without Cardiovascular Disease: Pooled Analyses of 4 Controlled Trials
Source: Am J Gastroenterol. 2021 May 25;116(8):1601–11. doi: 10.14309/ajg.0000000000001313 (PMC8315186; doi:10.14309/ajg.0000000000001313)
Supplement: SUPPLEMENTARY MATERIAL [file acg-116-1601-s002.pdf]

Supplemental Figure 2: Complete, considerable, and somewhat relief of IBS symptoms at each week (all women)

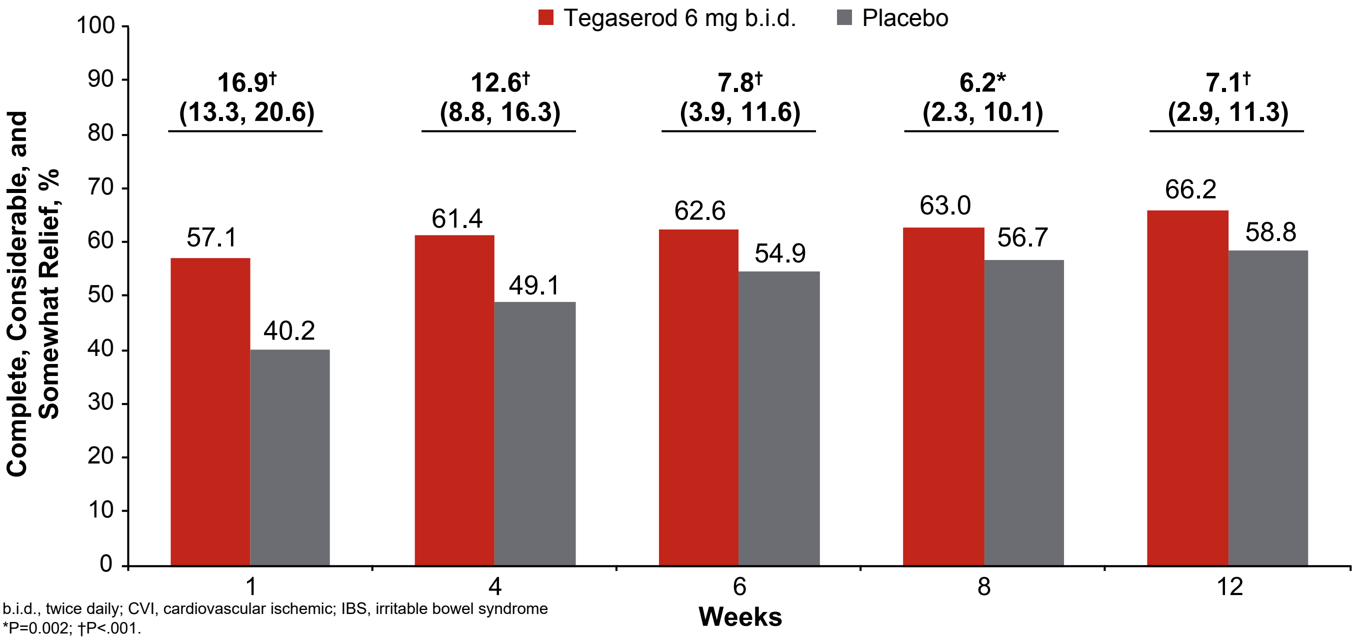

b.i.d., twice daily; CVI, cardiovascular ischemic; IBS, irritable bowel syndrome

*P=0.002; †P<.001.
