# Supplementary material for: Tegaserod for Irritable Bowel Syndrome With Constipation in Women Younger Than 65 Years Without Cardiovascular Disease: Pooled Analyses of 4 Controlled Trials
Source: Am J Gastroenterol. 2021 May 25;116(8):1601–11. doi: 10.14309/ajg.0000000000001313 (PMC8315186; doi:10.14309/ajg.0000000000001313)
Supplement: SUPPLEMENTARY MATERIAL [file acg-116-1601-s003.pdf]

Supplemental Figure 3A: Abdominal pain responder, 12 weeks (all women)

Study	Tegaserod n/N (%)	Placebo n/N (%)	OR (95% CI), *P*-value
**301**	18/237 (7.6)	11/235 (4.7)	1.73 (0.79–3.81), .173
**307**	15/228 (6.6)	14/226 (6.2)	1.09 (0.51–2.31), .826
**351**	25/222 (11.3)	15/226 (6.6)	1.77 (0.91–3.46), .091
**358**	268/766 (35.0)	212/746 (28.4)	1.35 (1.09–1.67), **.006**
**Pooled**	326/1453 (22.4)	252/1433 (17.6)	1.38 (1.14–1.67), **<.001**

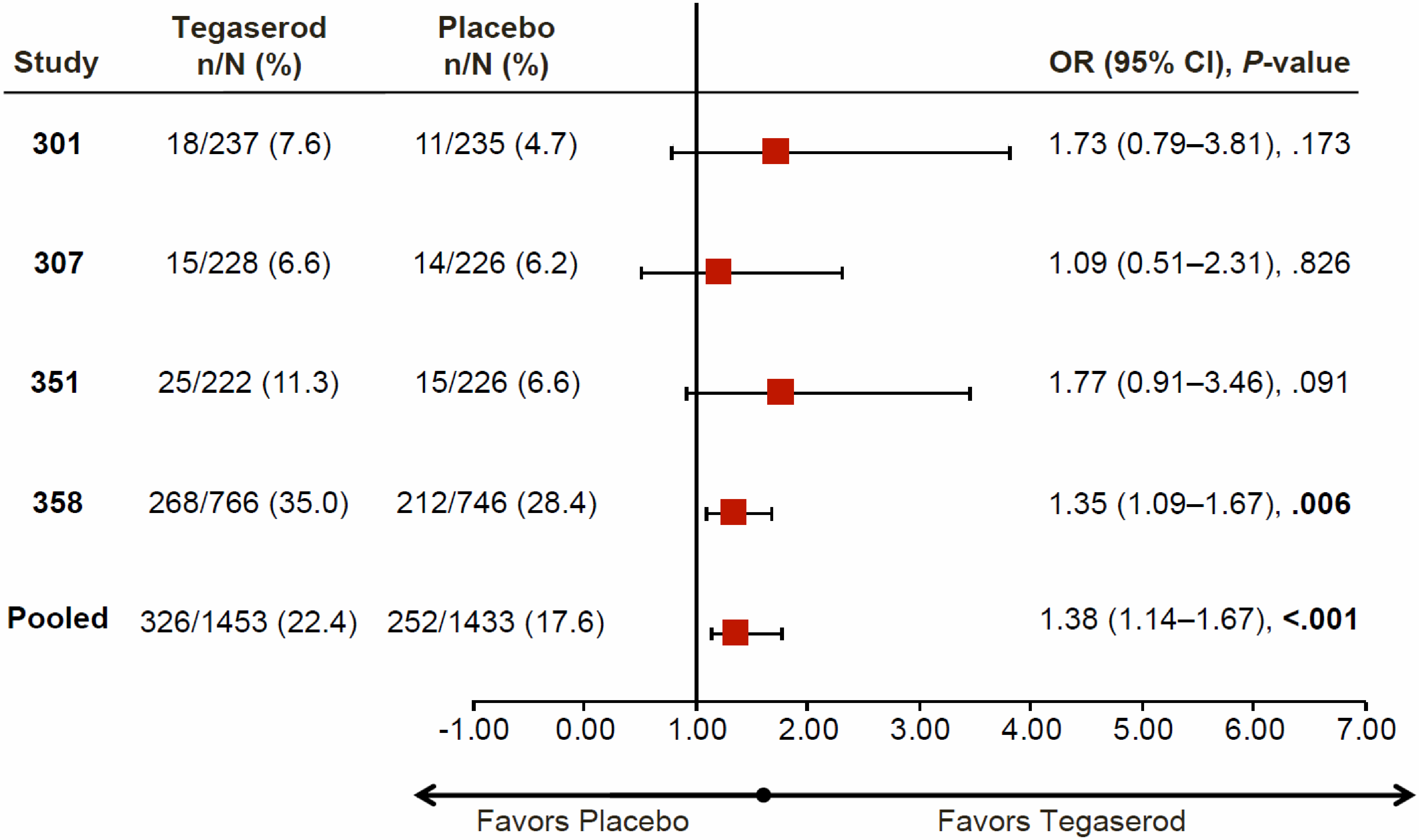

CI, confidence interval; OR, odds ratio.
Abdominal pain response defined as a ≥2-point improvement on a 7-point Likert scale.
