# Supplementary material for: Tegaserod for Irritable Bowel Syndrome With Constipation in Women Younger Than 65 Years Without Cardiovascular Disease: Pooled Analyses of 4 Controlled Trials
Source: Am J Gastroenterol. 2021 May 25;116(8):1601–11. doi: 10.14309/ajg.0000000000001313 (PMC8315186; doi:10.14309/ajg.0000000000001313)
Supplement: SUPPLEMENTARY MATERIAL [file acg-116-1601-s004.pdf]

Supplemental Figure 3B: Bloating responder, 12 weeks (all women)

Study	Tegaserod n/N (%)	Placebo n/N (%)	OR (95% CI), *P*-value
**301**	15/238 (6.3)	12/235 (5.1)	1.22 (0.55–2.69), .626
**307**	15/228 (6.6)	17/226 (7.5)	0.87 (0.42–1.79), .706
**351**	21/222 (9.5)	14/226 (6.2)	1.57 (0.78–3.17), .207
**358**	252/766 (32.9)	188/746 (25.2)	1.45 (1.16–1.81), **.001**
**Pooled**	303/1454 (20.8)	231/1433 (16.1)	1.39 (1.14–1.69), **.001**

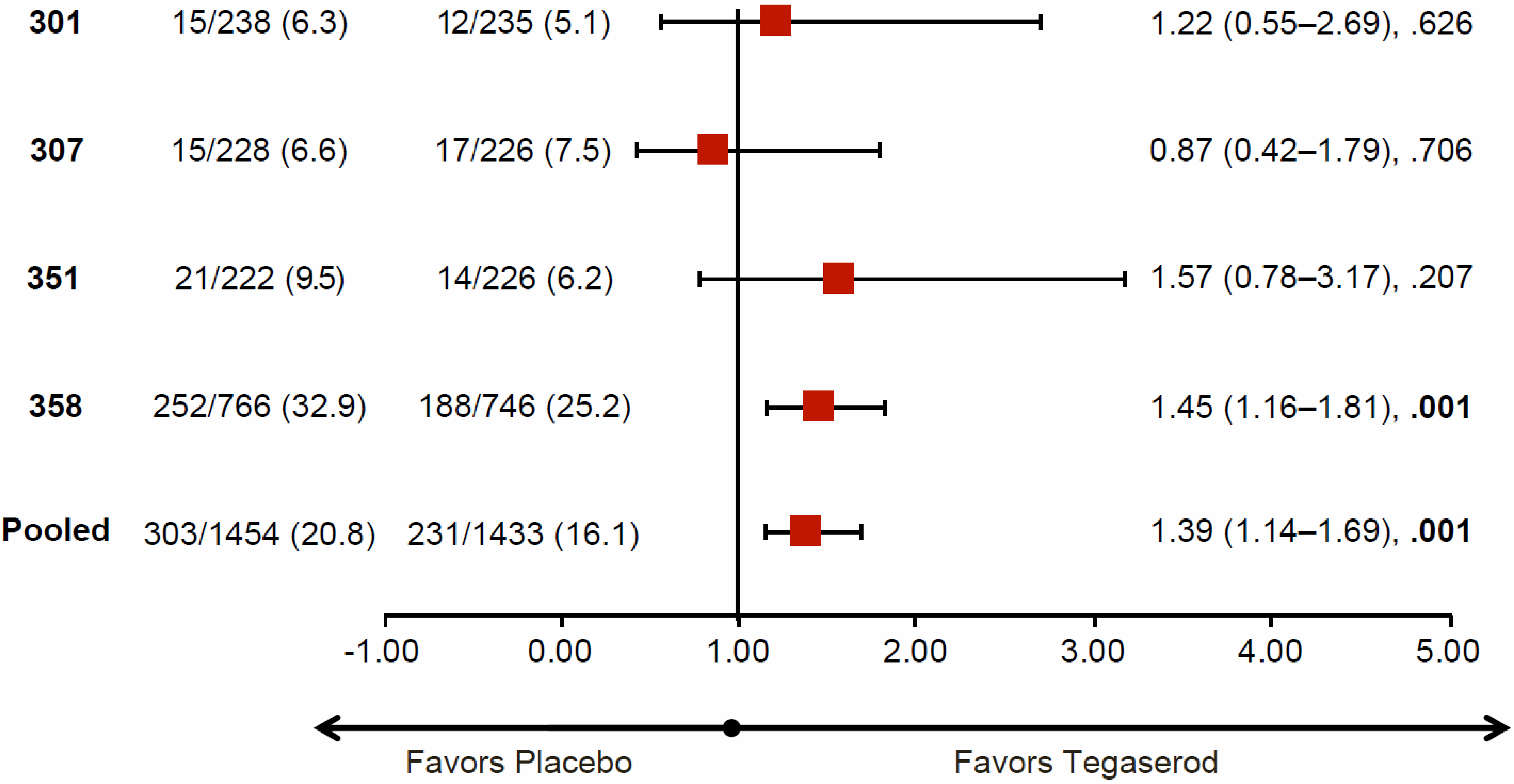

CI, confidence interval; OR, odds ratio.
Bloating response defined as a ≥2-point improvement on a 7-point Likert scale.
